# CONSTRUCTED FAMILY SYSTEMS FOR AGING LGBTQ ADULTS PLANNING FOR ELDER CARE

**DOI:** 10.1093/geroni/igz038.1112

**Published:** 2019-11-08

**Authors:** Lisa A Hollis-Sawyer, and Laura Tejada

**Affiliations:** Northeastern Illinois University, Chicago, Illinois, United States

## Abstract

Examining the intersectionality of sexuality, gender, and age, Fredriksen-Goldsen (2017) argued that the aging LGBTQ community is a marginalized aging group, and the lack of attention to their social and other support needs are creating negative aging trajectories for those who have experienced lifespan issues of disenfranchisement and unfair treatment affecting their health and care planning. The effect of a family can be a complex one and is further determined by social class, race, ethnicity, gender, and region. Allen and Roberto (2016) suggested that the LGBT adults may have three different types of family experiences, ranging from their “family of origin” (biological family) to “family of choice” (created kinship family). Oswald (2002) presented research on resilience in constructed family systems for aging gays and lesbians and identified two underlying processes toward establishing and maintaining family resilience: intentionality relates to the intentional actions of the older adult to establish and maintain the chosen family system while redefinition involves using language and symbols to reinforce their social support network. This idea of “doing family” is critical to comprehend from LGBTQ family members’ perspectives for eldercare planning. Social support needs for changing family dynamics begins in middle adulthood among aging gays and lesbians (Barker et al., 2006). Hypothetical case studies (e.g., sibling dynamics) will be discussed with implications for support and intervention programs to assist in the eldercare decision making of this growing aging subpopulation. Resource tools will also be presented to help achieve positive eldercare outcomes in this process of “doing family.”

